# Ophthalmic Formulations for the Treatment of Allergic Conjunctivitis and Their Effect on the Ocular Surface: A Review of Safety and Tolerability Assessments in Clinical Trials

**DOI:** 10.3390/jcm13226903

**Published:** 2024-11-16

**Authors:** Tatiana Suárez-Cortés, Ana Gonzalo, Eider Arana, Virginia Guillén, Noelia Andollo

**Affiliations:** 1 Research, Development and Innovation Department (R&D+I Department), FAES Farma, 48940 Leioa, Spain; agonzalo@faes.es (A.G.); earana.1@alumni.unav.es (E.A.); 2 Department of Cell Biology and Histology, School of Medicine and Nursing, University of the Basque Country, 48940 Leioa, Spain; noelia.andollo@ehu.eus; 3 Department of Neuroscience, Faculty of Medicine and Nursing, University of the Basque Country, 48940 Leioa, Spain; virginia.guillen@ehu.eus; 4 Biobizkaia Health Research Institute, 48903 Barakaldo, Spain

**Keywords:** ocular allergy, allergic conjunctivitis, homeostasis, dry eye disease, dysfunctional tear syndrome, antihistamines, ocular surface, tear film

## Abstract

Allergic conjunctivitis (AC) is the most common allergic eye disorder. Antiallergic eyedrops are the first line of pharmacological treatment. However, the application of antiallergic eyedrops can potentially alter tear homeostasis and affect the ocular surface, which may result in iatrogenic diseases such as dye eye disease (DED). Long-term treatment of AC with eyedrops containing preservatives and other components may increase the risk of DED and ocular surface damage. Here, we examined 20 clinical trials published during the past ten years with antihistamine ophthalmic formulations in the treatment of AC, to evaluate the extent of evidence about their safety and tolerability. Remarkably, we find that most trials lack an evaluation of the critical ocular surface parameters, such as tear film break-up time, tear volume, corneal and conjunctival damage, and inflammation, to properly assess the state of the ocular surface state after prolonged treatment. There is a need to increase awareness of the use of specific formulations that do not increase the risk of iatrogenic DED.

## 1. Introduction

Ocular allergy (OA) includes a set of different clinical entities considered ocular surface hypersensitivity disorders [1,2]. Allergic conjunctivitis (AC), the most common form of OA, is an immunological inflammatory process of the anterior surface of the eye that affects the eyelid, conjunctiva, and cornea and is mainly associated with an IgE-mediated immediate hypersensitivity response [1]. AC is usually bilateral, and the main symptom is intense ocular itching, which is reported by more than 75% of patients seeking treatment. Other signs and symptoms of AC include epiphora, hyperemia, micropapillae, conjunctival chemosis, and thin mucous discharge [3]. The health and normal function of the ocular surface depends on a stable and sufficient tear film. Consequently, any alteration in the volume or quality of the tear film can trigger dysfunctional tear syndrome, a chronic condition affecting the ocular surface. Dysfunctional tear syndrome includes multiple subtypes, such as dry eye disease (DED) and associated tear film disorders [4].

Although AC and DED are different entities with distinct etiologies, signs and symptoms such as discomfort, ocular redness, and itchiness are common to both, and a significant proportion of patients present both OA and DED simultaneously [5]. While these diseases may overlap in symptoms, the therapeutic approach is different. Therefore, differentiation between both pathologies is crucial to avoid therapeutic errors [6]. In DED management, the choice of the most suitable treatment should be made based on appropriate tests to confirm the diagnosis, followed by disease severity and etiological subtyping determination [7]. The goal of DED treatment is to restore homeostasis of the ocular surface by breaking the vicious cycle of the disease, and treatment of DED usually requires long-term instead of short-term treatment. Management commonly begins with conventional available therapies, such as over-the-counter lubricants, and can progress to more advanced therapies for more severe forms of DED [8]. Depending on the typology, there are treatments for tear insufficiency, which include tear replacement approaches (artificial tear substitutes for aqueous or lipid supplementation, biological tear substitutes, and other agents as mucolytics), tear conservation approaches (punctual occlusion), and tear stimulation approaches (topical secretagogues) [8,9]. In the case of OA, the management approach for both acute and chronic forms of OA start with allergen identification, followed by non-pharmacological treatments (allergen avoidance and hygiene measures); as the disease progresses, pharmacological treatments are recommended (antihistamines, mast cell stabilizers, dual-action agents, and corticosteroids) [10].

Eye drops for the treatment of DED are always evaluated for their effects on the ocular surface, tear film stability and epithelial barrier homeostasis [11]. The clinical overlap with OA suggests a complex interaction of mechanisms that involve the immune, endocrine, and nervous systems [12,13]. Artificial tears frequently used by patients with DED can improve symptoms in all clinical varieties of AC [13]. Also, it has become evident that alterations of tear film homeostasis and the epithelial barrier that can occur in OA can lead to DED and that people with DED can develop OA as a result of ocular surface inflammation [13,14,15,16]. Several tear inflammatory biomarkers are common for DED and OA [6,17]. Due to the overlapping symptoms between DED and OA, differential diagnosis can be challenging, and it is critical that clinicians correctly identify both entities [18].

Antiallergic eyedrops that are not well formulated to preserve ocular surface homeostasis can induce tear film disruption and exacerbate DED in predisposed patients. Iatrogenic ocular surface damage may be triggered when symptoms of AC are treated with antiallergic formulations that decrease tear production and/or alter the composition of tears. The presence of preservatives in antiallergic ophthalmic solutions contributes to DED by altering the tear film and causing epithelial barrier dysfunction [13,19]. In turn, the treatment of DED requires moisturizing and lubricating components, which are often missing in AC treatments. The components of eyedrop formulations for DED (such as ‘artificial tears’) are usually extensively evaluated to assess their impact on the tear film homeostasis [11,20,21,22,23,24]. However, it is unclear if the clinical trials of drugs for treating OA address tear film homeostasis assessment. For this reason, this review aimed to examine the available evidence of tear film and ocular surface safety assessment in recently published clinical trials of antiallergic (or antihistamine) ophthalmic formulations for the treatment of AC.

## 2. Clinical Trials of Ophthalmic Formulations for AC: Safety and Tolerability

A systematic review of randomized controlled trials (RCTs) of ophthalmic antihistamine formulations (N = 30) published from 1946 to 2014 concluded that, although the trials mainly only evaluated the short-term effects (<8 weeks), they appeared to be safe and well tolerated [25]. To extend the safety analysis to recently developed ophthalmic formulations of antihistamines for the treatment of AC, we searched and reviewed controlled trials published in the past ten years (2014 to 2023 inclusive; see Supplementary Materials for details). The search was conducted in PubMed in January 2024. The results are shown in Table 1.

The search found data in 20 controlled trials for formulations containing antihistamines for treatment of AC: alcaftadine [26,27,28,29,30,31], bepotastine [28,30,32], bilastine [33,34,35], cetirizine [36], emedastine [37], epinastine [38,39], ketotifen [35,38,40,41,42,43], and olopatadine [26,28,29,31,32,37,38,39,40,42,43,44,45]. Olopatadine 0.1% or 0.2% was analyzed in 13 controlled trials, followed by alcaftadine 0.25% and ketotifen 0.025%, both evaluated in 6 RCTs each.

**Table 1:** Randomized clinical trials of topical antiallergic eyedrops evaluating safety, tolerance, and/or comfort.

Author, Year, Reference	Design, Number of Patients (Active Treatment)	Drug Concentration, [Commercial Name]	Exposure	Safety/Tolerance Evaluation	Main Safety/Tolerance Conclusions
Marini, 2023 [32]	Prospective, multicenter, randomized, double-blind, controlled, parallel-group. Bepotastine, N = 48 Olopatadine N = 49	- Bepotastine 1.5% [Traler^®^ LC, Poen Laboratories, Argentina] - Olopatadine 0.2% [Patanol^®^ S, Alcon Laboratories, Argentina]	8 weeks, OD	- Conjunctival impression cytology - AEs	Non-preserved bepotastine 1.5% exhibits lower cytotoxicity than preserved olopatadine 0.2%
Logan, 2023 [40]	Randomized, single-center, patient-blind.	- Olopatadine 0.7% [Pataday^®^ Once Daily Relief Extra Strength; Alcon, Fort Worth, TX] - Ketotifen 0.035% [Alaway^®^, Bausch + Lomb, Bridgewater, NJ]	Single administration, 2 min	- Comfort (VAS)	Olopatadine 0.7% was initially more comfortable than ketotifen 0.035%
Kuna, 2023 [33]	Multi-center, international, randomized, double blind, placebo-controlled, parallel-group N = 218	- Bilastine 0.6% [Bilaxten^®^, FAES farma SA, Leioa, Spain]	8 weeks, OD	- AEs - Tolerability (VAS) - Comfort (VAS)	Bilastine 0.6% (preservative-free) is safe and well tolerated after long-term administration and exposure
Gomes, 2023 [34]	Single-center, double-masked, randomized Bilastine 0.2%, N = 30 Bilastine 0.4%, N = 30 Bilastine 0.6%, N = 31	- Bilastine 0.2%, 0.4%, and 0.6% [FAES farma SA, Leioa, Spain]	CAC model (single administration, 16 h)	- AEs - Comfort (Drop comfort scale, drop comfort questionnaire)	Bilastine 0.6% (preservative-free) is safe and well tolerated
Gomes, 2023 [35]	Multicenter, double-masked, randomized Bilastine 0.6%, N = 91 Ketotifen 0.025%, N = 90	- Bilastine 0.6% [Bilaxten^®^, FAES farma SA, Leioa, Spain] - Ketotifen 0.025%	CAC model (single administration, 16 h)	- AEs	Bilastine was safe and well tolerated. Mean drop comfort scores were significantly better for bilastine than for ketotifen immediately upon instillation (*p* < 0.05) and similar to those of the vehicle.
Fujishima, 2021 [31]	Single-center, placebo-, and comparator-controlled, randomized N = 224	- Alcaftadine 0.25% [AGN-229666, Lastacaft^®^] - Olopatadine 0.1% [Pataday^®^]	CAC model (single administration, 16 h)	- AEs	Alcaftadine 0.25% is safe and well tolerated
Ayyappanavar, 2021 [30]	Prospective, observer-masked, comparative, randomized Alcaftadine, N = 60 Olopatadine, N = 60 Bepotastine, N = 60	- Alcaftadine 0.25% [NR] - Olopatadine 0.2% [NR] - Bepotastine 1.5% [NR]	2 weeks, (alcaftadine and olopatadine, OD; bepostatine, BID)	- AEs	All three medications are safe
Çavdarli, 2020 [44]	Randomized, single-center N = 39	- Olopatadine 0.1% [Patanol^®^, Alcon Research Ltd.]	Single administration, 45 min	- Pentacam topography	Olopatadine 0.1% does not change corneal topography or anterior chamber parameters. It causes a statistically significant increase in pupil diameter.
Nakatani, 2019 [29]	Single-center, randomized, double-masked, vehicle and active-controlled Alcaftadine, N = 47 Olopatadine, N = 49	- Alcaftadine 0.25% [NR] - Olopatadine 0.1% [Patanol^®^, Alcon Laboratories, Inc., Fort Worth, TX, USA]	CAC model (single administration, 8 h)	- AEs	Alcaftadine and olopatadine are safe and well tolerated
Malhotra, 2019 [36]	Three studies: Phase I (prospective, single-center, open-label), two Phase III (multi-center, randomized, double-masked, vehicle-controlled, parallel-group) N = 341	- Cetirizine 0.24% [Zerviate^®^, Akorn, Inc., Lake Forest, Illinois, USA]	6 weeks (BID, TID)	- Comfort (VAS)	Cetirizine 0.24% dosed BID or TID is safe and well tolerated
Leonardi, 2019 [41]	Single-masked, randomized, multicenter study N = 81	- Ketotifen 0.025% [Zaditen Oftabak, Thea, France], or 0.05% [Ketoftil, Farmigea, Italy]	3 weeks (OD)	- Tolerability	Ketotifen 0.025% (preservative-free) is better tolerated than ketotifen 0.05% in seasonal AC
Dudeja, 2019 [28]	Prospective, observer-masked, randomized Olopatadine, N = 15 Bepotastine, N = 15 Alcafatadine, N = 15	Olopatadine 0.1% [NR] Bepotastine 1.5% [NR] Alcafatadine 0.25% [NR]	4 weeks (BID)	- Tolerability	All three drugs are well tolerated.
Cao, 2019 [37]	Randomized Olopatadine, N = 795 Emedastine, N = 805	- Olopatadine 0.2% [Pataday^TM^, Alcon Laboratories, Inc., USA] - Emedastine [Emadine, Alcon Laboratories, Inc., USA]	2 weeks (OD), with 3-month FU	- Conjunctival impression cytology - AEs	No differences between drugs in conjunctival impression cytology assessments
Anusha, 2019 [38]	Randomized, prospective, comparative Olopatadine, N = 30 Ketotifen, N = 30 Epinastine, N = 30	- Olopatadine [NR] - Ketotifen [NR] - Epinastine [NR]	4 weeks (BID)	- AEs	Olopatadine is better tolerated than ketotifen and epinastine
Patel, 2018 [42]	Randomized Olopatadine 0.1%, N = 55 Ketotifen 0.025%, N = 54	- Olopatadine 0.1% [Winolap^®^, Sun, Avesta] - Ketotifen 0.025% [Albalon^®^, Allergan]	4 weeks (olopatadine, BID; ketotifen, 4 times daily)	- QoL	Both medications improved the QOL to a similar extent
McLaurin, 2015 [45]	Multicenter, double-masked, phase 3, randomized Olopatadine 0.77%, N = 98 Olopatadine 0.2%, N = 99 Olopatadine 0.1%, N = 99	- Olopatadine 0.77% [IP], 0.2% [Pataday^®^], 0.1% [Patanol^®^]	CAC model (single administration, 24 h)	- Slit-lamp biomicroscopy	Olopatadine 0.77% is safe
Ciolino, 2015 [27]	Two double-masked, multicenter, placebo-controlled, randomized N = 96	- Alcaftadine 0.25% [Lastacaft^®^, Allergan, Inc., Irvine, CA, USA]	CAC model (single administration, 7 min)	- AEs	Alcaftadine 0.25% is well tolerated
Mortemousque, 2014, [43]	Randomized, investigator-masked, comparative Ketotifen, N = 38 Olopatadine, N = 37	- Ketotifen 0.025% [NR] - Olopatadine 0.1% [NR]	4 weeks (daily administration)	- Patient-assessed symptoms - Physician-assessed ocular signs	Ketotifen 0.025% (unpreserved) presents slightly better ocular tolerance than olopatadine (preserved)
McLaurin, 2014 [26]	Two double-masked, multicenter, active- and placebo-controlled, randomized Alcaftadine, N = 96 Olopatadine, N = 95	- Alcaftadine 0.25% [Lastacaft^®^, Allergan, Inc., Irvine, CA, USA] - Olopatadine 0.2% [Pataday^®^, Alcon Laboratories, Inc., Fort Worth, TX, USA]	CAC model (single administration, 24 h)	- AEs	No safety concerns
Fujishima 2014, [39]	Randomized, placebo controlled Epinastine, N = 87 Olopatadine, N = 43	- Epinastine 0.05% [Alesion^®^, Santen Pharmaceutical] - Olopatadine 0.1% [Patanol^®^, Alcon Laboratories]	CAC model (single administration, 8 h)	- AEs	Epinastine has a good safety profile

AC, allergic conjunctivitis; AEs, treatment emergent adverse effects; BID, twice a day; CAC, conjunctival allergen challenge; IP, investigative product; NR, not reported; OD, once daily; TID, three times a day; VAS, visual analogue scale.

### 2.1. Evaluation of Safety

Of the 20 clinical trials, 7 followed the conjunctival allergen challenge (CAC) model to investigate short-term efficacy and safety (up to 24 h). These trials investigated only immediate or short-term adverse effects. The rest described longer exposures, in the range of days or weeks, with some of the longest trials performed with bepotastine and bilastine evaluating safety and tolerability after eight weeks of daily administration [32,33].

The most frequent patient-reported adverse effects in trials evaluating more than one week of treatment were mild to moderate irritation, dry eye, and eye discharge. Dry eye and irritation have been frequently described as adverse reactions in trials of antiallergy eyedrops. A trial of a formulation of cetirizine described mild hyperemia in >10% of patients [36]. Overall, the trials suggest that topical administration of antihistamines for AC is safe and well tolerated.

Notably, information on eye surface safety was absent in most RCTs. None of the trials evaluated parameters of the ocular surface homeostasis related to tear function, such as the OSDI scale of symptoms, tear volume, tear flow, TBUT, tear osmolality, or tear and epithelial biomarkers. Two studies used conjunctival impression cytology to assess parameters related to ocular surface health, such as corneal epithelial defect, squamous metaplasia, goblet cell density, superficial punctate keratopathy (SPK), and corneal markers [32,37]. Only one trial evaluated corneal topography after the administration of a solution of olopatadine 0.1% [44].

One trial evaluated the quality of life after one month of administration of olopatadine 0.1% and ketotifen 0.025% [42].

### 2.2. Assessment of Tolerability and Comfort

Many of the trials investigated tolerance and/or comfort during drug administration, usually using patient-reported visual analog scales [28,33,35,36,40,41]. These evaluations revealed that most formulations were well tolerated.

Tolerability can have a major negative impact on adherence to treatment. For example, olopatadine, bepotastine, and alcaftadine are antihistamines that have been shown to have mast-cell stabilizing properties and, therefore, are supposed to target both the early and the late-phase allergy mediators. Generally, the onset of action of ophthalmic antihistamine or antiallergy formulations is fast, but this effect on late-phase mediators can take about two weeks to achieve full efficacy.

If the patient needs to instill topical antihistamines for an extended period of time, their tolerability becomes important to improve adherence [46]. Regarding treatment adherence, a study in Japan found that only about 10% of patients with DED instilled eyedrops at the frequency specified in the package insert [47]. Unfortunately, there are no studies of adherence to antiallergic formulations for AC. It has been speculated that solutions that require only once-a-day administration can improve adherence [35].

### 2.3. Impact of Preservatives, pH, Osmolarity, and Lubricants on the Ocular Surface

Although there are many studies reporting the efficacy of antihistamine ophthalmic formulations for the treatment of AC, few of them have focused on their impact on those aspects related to a potential compromise of tear film homeostasis and ocular surface integrity. In the following sections, we review the evidence in clinical trials of the effects of preservatives, non-physiological pH, or salts, on the ocular surface [13,16,18,46].

#### 2.3.1. Effects of Preservatives

The presence or absence of preservatives was not explicitly indicated in all the included studies. As described in a recent review focusing on this, most of the available antihistamine ophthalmic formulations in multidose bottles contain benzalkonium chloride (BAK) as a preservative [48]. The deletereal effects of BAK on the ocular surface can be observed after exposures as short as seven days [49]. Only four of the included RCTs in this review evaluated preservative-free formulations: one with ketotifen [41], two with bilastine [24,26], and one with bepotastine [32].

A study comparing a once-daily non-preserved bepotastine 1.5% formulation versus a BAK-preserved olopatadine 0.2% found, in conjunctival impression cytology, significant differences in the histology of the conjunctival epithelium between the treatment groups (Nelson scale, *p* = 0.001) [32]. After 60 days of treatment, the normal conjunctiva in the BAK-preserved olopatadine group decreased by 27.4%, whereas in the non-preserved bepotastine group, there was an improvement of 20.5% (*p* = 0.006). At the end of the trial, some conjunctival cytologies in the olopatadine group presented epithelial metaplasia. However, in this study, the olopatadine 0.2% ophthalmic solution was preserved with BAK 0.01%, one of the highest concentrations of BAK used to prepare eyedrops. Although no causality could be derived from this study, the authors suggested that is possible that BAK in the olopatadine formulation could be responsible for the toxic effects on the epithelium [32].

#### 2.3.2. Assessment of pH Impact

Another parameter for which the included clinical trials provide very little information is the pH of the ophthalmic solutions tested. The administration of ophthalmic formulations with acidic pH values, as is the case of some formulations, or alkaline values generates a certain degree of discomfort and ocular irritation that is accompanied, in turn, by an increase in tear secretion as a defense mechanism to restore normal physiological conditions [50]. The excessive tearing generated, in turn, favors the loss of the drug and, therefore, reduces the bioavailability and therapeutic effect of the product [51]. In this regard, a recent study comparing initial comfort during the administration of olopatadine 0.7% (pH 7.2) and ketotifen 0.035% (pH between 4.4 and 6.0) suggested that, as the olopatadine solution was at a pH much closer to that of human tears than the ketotifen solution, this was the reason for its better initial comfort scores [40].

#### 2.3.3. Evaluation of Osmolarity

Another critical aspect related to the tolerability and safety of the ocular surface is osmolarity [52]. Hyperosmolar topical formulations have been shown to alter tear osmolarity, generating a sensation of discomfort and potentially causing damage to the ocular surface and inflammation [20,52,53]. However, none of the formulations tested in the RCTs evaluated mentioned osmolarity as a factor of safety or tolerability. Large differences in osmolarity values are found within the main antiallergic eyedrops. For example, in a recent in vitro comparative study of nine of the most common commercially available antihistamine formulations, it was found that eight were close to tear osmolarity, in a range from 245 to 331 mOsm/L, except for Bilina^®^ (Esteve Pharmaceuticals, Barcelona, Spain; levocabastine hydrochloride 0.05%), an ophthalmic suspension presenting a hyperosmolar value of 1038 mOsm/L [54].

Previous in vivo studies in mice showed that hyperosmolarity stimulates the expression and production of inflammatory mediators such as IL-1beta, TNF-alpha, and MMP-9, and activates c-jun N-terminal kinase (JNK), extracellular-regulated kinases (ERKs), mitogen-activated protein kinase (MAPK) and p38 signaling pathways on the ocular surface. This study suggested that hyperosmolar stress promotes ocular surface inflammation in a similar way as in dry eye. Therefore, attention to this parameter should be taken [55].

#### 2.3.4. Effect of Moisturizing Additives

Topical formulations often contain additives such as hydrophilic polymers to increase viscosity, improve retention time, and promote hydration and lubrication of the ocular surface during blinking [48]. The increase in viscosity can decrease the drainage rate, prolong precorneal contact time and so increase ocular absorption and drug bioavailability in the target tissue [51]. Some of these additives include hyaluronic acid, dextran 70, gelatin, poloxamer 407, polyvinylpyrrolidone, cellulose derivatives (methylcellulose, hypromellose, and carmellose), and acrylic acid derivatives (carbopol) [48].

The RCTs reviewed did not, except in some cases, explicitly mention the presence or absence of these excipients as a factor that could be relevant to the results of the studies. The use of hyaluronic acid as an excipient in ophthalmic formulations has been amply documented. It has been shown to reduce tear washing and promote the stability of the tear film, and, in the RCTs performed with the bilastine 0.6% preservative-free formulation, it has been shown to provide symptom relief to patients and a feeling of comfort [20,56,57]. In a comparative study, ophthalmic formulations containing higher-MW hyaluronic acid, characterized by a low polydispersion index, could present better hydration, lubrication, re-epithelialization, and anti-inflammatory properties that protect the ocular surface and, therefore, have greater therapeutic potential [20,58].

The potential use of combining carboxymethylcellulose and hyaluronic acid polymers [59], carboxymethylcellulose 1.0% and glycerin 0.9% [60], a combination of hydroxypropyl guar and hyaluronic acid [61], ectoine [62,63], or trehalose [64] has been documented.

In turn, the combination of hyaluronic acid with antiallergic compounds in ophthalmic formulations may provide potential benefits due to its capacity to increase bioavailability and protective effects against conjunctival dehydration [10].

#### 2.3.5. Weighing the Effect of Buffering Agents

Buffering agents are usually part of ophthalmic formulations, and include molecules such as acetic, boric, and hydrochloric acid, potassium or sodium bicarbonate, phosphate, or citrate. Phosphate is a commonly used buffer in eyedrops because its high buffering capacity stabilizes the pH level at 7.4 [65]. However, eyedrops containing phosphates have been identified as possible generators of calcium deposits in the cornea and a recent review concluded that about 40% of glaucoma medications and approximately 60% of corticosteroid and antihistaminic medications had a phosphate concentration higher than the physiologic tear phosphate level of 1.45 mmol/L [66]. Evaluation of 37 commercial antiglaucomatous eyedrops commercialized in Spain found that in all the eyedrops the concentration of phosphates exceeded that of the tear film [67]. Also, Opatanol^®^ (olopatadine 1 mg/mL) contains 3.34 mg/mL (3340 ppm) of disodium hydrogen phosphate dodecahydrate as a buffering agent. Unfortunately, none of the clinical trials reviewed here indicated the concentration of phosphates or other buffer components [68].

A recent comparative in vitro study measured the phosphate concentration (PO_4_^3^) of nine commercially available antiallergic ophthalmic formulations for the treatment of AC. The study found that five of the nine evaluated formulations contained phosphates in a wide range. Pazeo^®^ (Alcon Research, Fort Worth, TX, USA; olopatadine hydrochloride 7.7 mg/mL) contains 1.8 ppm, followed by Pataday^®^ (Alcon Laboratories, Fort Worth, TX, USA; olopatadine hydrochloride 2.2 mg/mL) with 3.21 ppm, and Opatanol^®^ (Novartis, Barcelona, Spain; olopatadine 1 mg/mL) containing 3324 ppm, Tebarat^®^ (Salvat, Barcelona, Spain; azelastine hydrochloride 0.5 mg/mL) with 3.66 ppm; and Bilina^®^ (Esteve Pharmaceuticals, Barcelona, Spain; levocabastine hydrochloride 0.5 mg/mL) contains the highest phosphate concentration (9207 ppm). Besides these, three formulations classified as without phosphates contain <0.6 ppm, including Afluon^®^ (Mylan Pharmaceuticals, Barcelona, Spain; azelastine hydrochloride 0.5 mg/mL), and single-dose and multidose Zaditen^®^ (Théa, Barcelona, Spain; ketotifen fumarate 0.25 mg/mL) formulations; whereas only Bilaxten^®^ (Faes Farma, Bizkaia, Spain; bilastine 6 mg/mL) contains <0.3 ppm and was classified as phosphates-free [54].

## 3. Towards the Preservation of Ocular Surface Homeostasis in AC

This review, focused on the safety and tolerance parameters of antiallergic eyedrop formulations’ clinical trials published in the past ten years, reveals that few of them investigated their possible impact on the ocular surface. Given the high prevalence of the comorbidity of AC and DED, a better assessment of ocular surface parameters in clinical trials of antihistamine formulations seems required. This would involve relatively simple procedures, such as administering the Ocular Surface Disease Index (OSDI) questionnaire or Schirmer’s test, and taking measurements of the tear break-up time (TBUT) or the tear meniscus height (TMH). It should also be desirable to evaluate the expression of inflammatory biomarkers in tears, as well as the state of the corneal and conjunctival epithelia and the expression of inflammatory mediators by impression cytology [69]. All of these tools are routinely applied in the development of eyedrops formulated for DED treatment [11,70]. A table with ocular surface assessment parameters and methods is shown in Supplementary Table S2 [7,71]. Also, evaluation of the safety and tolerability of ophthalmic formulations for AC should have an adequate length, as long-term use of these drugs can have unexpected effects on the ocular surface. Although the maximum recommended time that these drugs should be administered without seeking medical advice is sometimes indicated in the prospectus, in other cases it is indicated that the treatment must continue while symptoms persist, or there is no indication of the maximum time of administration (e.g., with Opatanol^®^, Bilina^®^, Bentifen^®^, Emadine^®^, and Corifina^®^).

**Figure 1:**
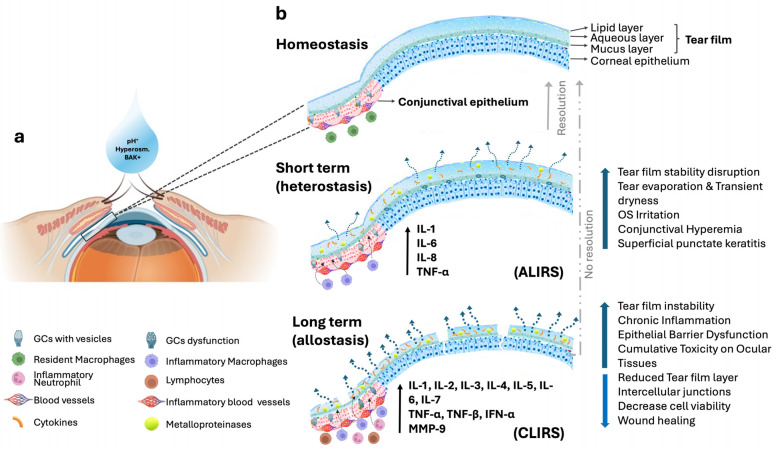
Representation of the potential short- and long-term effects of antiallergic eyedrops on the ocular surface homeostasis. (**a**) Schematic representation of the anterior segment of the eye. The box indicates a zoom of a section of the corneal and conjunctival epithelium overlayed with the tear film. (**b**) The upper panel is an illustration of homeostasis of the tear, corneal, and conjunctival epithelia. The middle panel represents the short-term time effects, in which a disruption of the tear film stability initiates an increase of the tear evaporation together with a transient dryness; irritation induces stinging or burning sensations upon instillation; at epithelial level, dilation of blood vessels induces conjunctival hyperemia and punctate epithelial erosions may occur, starting corneal epithelial alterations. The instability of the tear film and subjective discomfort are amongst the very early signs of ocular surface challenge [72]. In this state, an ALIRS is activated to temporarily manage inflammatory conditions and preserve the ability to restore homeostasis (Resolution). The lower panel represents the long-term effects, in which an alteration of tear composition and dynamics exacerbates symptoms of dryness and irritation; in addition, a chronic ocular surface inflammation by the sustained expression of cytokines and inflammatory molecules can lead to persistent discomfort, redness, and compromised visual health. Disruption of the epithelial barrier may increase susceptibility to microbial invasion and other ocular surface diseases. This established cumulative toxicity may induce progressive damage to ocular tissues, including thinning of the epithelium and impaired wound healing. When these alterations become constant, a CLIRS is established, and their resolution to return to a state of homeostasis is unlikely. ALIRS: acute leucocytic irritative response system; CLIRS: chronic leucocytic irritative response system. Illustration partially created with BioRender.

The use of antiallergic eyedrops that are not well adapted to preserve the homeostasis of the ocular surface may reduce the comfort of patients after instillation and preclude patients from correct treatment compliance. Moreover, the presence of preservatives in antiallergic eye drop formulations contributes importantly to the cytotoxic effects induced by these compounds and can have complex and profound short- and long-term effects (Figure 1). For example, BAK is a common preservative found in many ophthalmic solutions, including antiallergic eyedrops [1]. While these eyedrops are effective in managing allergic reactions, the presence of BAK can induce adverse effects on the ocular surface, affecting both tears and the epithelial tissues of the conjunctiva and cornea [2].

BAK can cause histopathological changes that initially remain asymptomatic but could then evolve into more severe stages of ocular surface disease. The successive administration of ophthalmic formulations with preservatives generates toxic and inflammatory effects on the ocular surface, which damage the epithelial cells of the cornea and conjunctiva, particularly in those patients whose surface is compromised. Furthermore, its frequent use has been associated with alterations of the precorneal film, such as instability of the tear film, observing, in patients with iatrogenic dryness, a tendency to aggravate the already existing problem. These changes can cause severe impairments if the ophthalmic formulation is administered in the long term [19]. In fact, BAK is used for developing DED in animal models to study its effects on the ocular surface, and to evaluate the preclinical efficacy of therapeutic treatments in these models [73]. Also, in other diseases, such as glaucoma, the onset of ocular surface diseases has been frequently reported in patients prescribed with benzalkonium chloride (BAK)-containing topical glaucoma medications for prolonged periods [74]. In this regard, a recent review indicated that 48–59% of patients with glaucoma experience symptoms of ocular surface disease, and 22–78% may exhibit clear clinical signs [75]. A study of patients that switched from BAK-preserved prostaglandin therapy to a preservative-free formulation of prostaglandin showed that, after only three months, there was a significant reduction in the rates of irritation/burning/stinging, itching, foreign body sensation, tearing, and dry eye sensation, with the same level of intra-ocular pressure control [76]. Therefore, understanding these undesired effects of BAK is crucial for optimizing treatment strategies and minimizing potential risks (Table 2).

The effects of BAK have been evaluated in several short-term pre-clinical and long-term clinical studies. A recent preclinical study comparing preserved and preservative-free antiallergic formulations in primary corneal and conjunctival epithelial cells found that preservative-free formulations, such as bilastine, single-dose ketotifen, and single-dose azelastine, resulted in higher cell survival rates than preserved antiallergic eye drops. This enhanced cell viability was attributed to the absence of preservatives, which prevented caspase-3/7-mediated apoptosis after 24 h of cell exposure [54]. Clinically, a trial comparing preservative-free and preserved levocabastine 0.05% ophthalmic solutions for preventing allergic conjunctivitis not only showed that the preservative-free version was more effective than the placebo and the preserved suspension, but that it also had fewer adverse events and received better drop sensation ratings [77]. Similarly, a very recent study compared the preservative-free bimatoprost 0.01% with BAK-containing bimatoprost 0.01% following a 12-week treatment period in patients with open angle glaucoma or ocular hypertension [78]. This study concluded that the preservative-free ophthalmic gel had the same efficacy in lowering intra-ocular pressure as the preserved gel but demonstrated less aggravation of conjunctival hyperemia after 6 or 12 weeks of treatment.

A recent model proposes a transition from ocular surface homeostasis (physiological balance) to heterostasis (a temporary condition or stage of cellular instability in response to environmental challenges), to subsequent allostasis (loss of homeostasis), which implies a new steady state of balance at a higher level of constant cell stress and inflammation [72]. In this model, temporary lubrication insufficiencies activate an acute leucocytic irritative response system (ALIRS) that can temporarily handle inflammatory conditions and retain the coping of the ocular surface (capacity of regaining and re-establishing homeostasis). However, when lubrication deficiencies become constant, chronic leucocytic irritative response systems (CLIRS) with inflammatory reactions are established. These transitional stages of the model would be equivalent to the short- and long-term physiological effects, respectively (Figure 1).

According to the physicochemical properties, an ophthalmic formulation is considered optimal for maintaining the integrity of the ocular surface when it has a physiological pH, is an iso- or hypo-osmolar solution, and excludes phosphates. Furthermore, it should provide hydration and lubrication to the ocular surface when it has adequate viscosity values, obtained by incorporating hydrophilic polymers into its composition. The use of preservative-free formulations is always advisable, especially in patients with greater exposure to high doses or prolonged treatments, or in those who suffer from chronic diseases, pre-existing or concomitant ocular surface disorders, and in those experiencing ocular surface-related side effects [5,19].

In addition to allergy, there are external factors that impact tear and ocular surface homeostasis, such as the excessive use of digital screens (digital asthenopia or computer vision syndrome) [79]. There is a correlation between the number of hours watching digital screens and the onset of symptoms of dry eye symptomatology [80], and the prevalence of dry eye in computer users can be as high as 50% [81]. In this regard, the use of hyaluronic acid has been shown to offer some protective effects in a recent study [82].

OA and DED are highly prevalent conditions and the most frequent reasons for ophthalmological consultation [83]. It has been proposed that ophthalmologists should evaluate the tear status of patients with AC (e.g., by performing a Schirmer test, TBUT, and tear film break patterns) to rule out the possibility of comorbid DED [15]. Similarly, the possibility of a history of allergic diseases should be investigated in patients with DED, and the findings of palpebral erythema and edema should be noted. A recent systematic review and meta-analysis of studies evaluating the prevalence of comorbidity of AC and DED showed that almost half of the patients with AC exhibit comorbid DED, and almost 20% of patients with DED may exhibit comorbid AC [15]. Other studies have also reported high rates of comorbidity, especially in Asian populations [84,85]. The relatively higher comorbidity rate of DED in patients with AC suggests that patients with AC may be predisposed to DED. There is an unmet medical need for validated biomarkers that could be used as biological tools for managing ocular surface diseases, such as DED and OA, and the early detection of alterations of ocular surface homeostasis to allow adopting a clinical decision to switch to harmless topical treatments [6]. High rates of DED have also been observed in children with AC [86,87,88]. Therefore, special attention should be paid to the maintenance of ocular surface homeostasis to avoid feedback from an AC–DED vicious circle, potentially created in situations of AC and DED comorbidity. Managing patients with ocular surface disease requires a multi-faceted approach focused on reducing ocular surface toxicity, improving tear film stability, and controlling inflammation. In those cases, switching to preservative-free medications, using supportive treatments for the ocular surface, and regular monitoring are key components of this strategy [75].

In summary, given the large number of treatments available for AC, there is a need to increase awareness of the use of eye drop formulations that do not increase the risk of iatrogenic DED, or prevent the progression of the DED from an acute to a chronic condition. Raising awareness would assist healthcare providers in prescribing or recommending the most suitable products, such as those that preserve the stability and integrity of the tear film, promote the homeostasis of the ocular surface, and avoid the toxic effects of preservatives. Increased demand for formulations that are safer for the ocular surface would incentivize pharmaceutical and healthcare companies to develop and promote products that align with these preferences.

In this regard, the most promising antiallergic eyedrops should ideally be formulations adapted to the physiological conditions of human tears, regarding pH, osmolarity, and viscosity to preserve the tear film stability; once-daily dosage, which could promote adherence and compliance to treatment; preservatives-free, to avoid any toxic effect on epithelial cells; phosphate-free, to avoid corneal calcification; be able to provide hydration to the ocular surface, containing in its formulation moisturizing additives such as dextran 70, gelatin, poloxamer 407, polyvinylpyrrolidone, cellulose derivatives (methylcellulose, hypromellose, and carmellose), and acrylic acid derivatives (carbo-pol); and providing re-epithelialization properties.

## Figures and Tables

**Table 2:** Short-term and long-term effects of BAK on the ocular surface.

Short-Term Effects	
Tear Film Stability Disruption	BAK has surfactant properties that can destabilize the tear film upon instillation. This disruption may manifest as increased tear evaporation, leading to transient dryness and discomfort.
Ocular Surface Irritation	Immediately after application, antiallergic eyedrops containing BAK can cause stinging or burning sensations on the ocular surface. This irritation is often attributed to BAK’s cytotoxic effects on epithelial cells.
Conjunctival Hyperemia	Short-term exposure to BAK can result in conjunctival hyperemia, characterized by dilated blood vessels on the surface of the eye. This redness is a common adverse reaction to BAK-containing eyedrops and typically resolves shortly after instillation.
Corneal Epithelial Damage	BAK has been associated with corneal epithelial toxicity, particularly in high concentrations or frequent usage. This can manifest as punctate epithelial erosions or superficial keratitis, contributing to symptoms of ocular discomfort and blurred vision.
**Long-Term Effects**	
Tear Film Instability	Long-term use of BAK-containing eyedrops can contribute to tear film instability by altering the composition and dynamics of tears. This instability may further perpetuate ocular surface damage and exacerbate symptoms of dryness and irritation.
Chronic Ocular Surface Inflammation	Prolonged use of antiallergic eyedrops with BAK may exacerbate chronic inflammation of the ocular surface. This inflammatory response can lead to persistent discomfort, redness, and compromised visual acuity over time
Epithelial Barrier Dysfunction	Disruption of the epithelial barrier function of both the conjunctiva and cornea. This impairment may increase susceptibility to microbial invasion and exacerbate ocular surface diseases, such as dry eye syndrome.
Cumulative Toxicity on Ocular Tissues	In the conjunctiva and cornea, cumulative toxic effects can lead to progressive damage and compromise ocular health. This includes thinning of the epithelium, reduced cell viability, and impaired wound-healing mechanisms.

## Supplementary Materials

The following supporting information can be downloaded at: https://www.mdpi.com/article/10.3390/jcm13226903/s1, Table S1: Summary of search terms; Table S2: Ocular surface assessment parameters and methods.
