# Supplementary material for: Ophthalmic Formulations for the Treatment of Allergic Conjunctivitis and Their Effect on the Ocular Surface: A Review of Safety and Tolerability Assessments in Clinical Trials
Source: J Clin Med. 2024 Nov 16;13(22):6903. doi: 10.3390/jcm13226903 (PMC11595245; doi:10.3390/jcm13226903)
Supplement: Supplementary file 1 [file jcm-13-06903-s001.zip › jcm-3252815-supplementary.pdf]

Supplementary Materials

for

Ophthalmic Formulations for the Treatment of Allergic Conjunctivitis and Their Effect on the Ocular Surface: A Review of Safety and Tolerability Assessments in Clinical Trials

by

Suárez-Cortés, T. *et al*.

Supplementary Table 1. Summary of search terms.

Keywords: ((allergic conjunctivitis) OR (ocular allergy) OR (eye allergy)) AND ((Conjunctivitis, Allergic*/drug therapy) OR (anti-allergic agents) OR (antihistamines) OR (antazoline) OR (emedastine) OR (levocabastine) OR (bepotastine) OR (alcaftadine) OR (epinastine) OR (azelastine) OR (ketotifen) OR (olopatadine) OR (bilastine) OR (cetirizine) OR (desloratadine) OR (ebastine) OR (fexofenadine) OR (levocetirizine) OR (loratadine)) NOT ((review) OR (meta-analysis) OR (systematic review)) AND ((topical) OR (ophthalmic))
Filter: 2014-2023
Filter: “clinical trial” OR “randomized controlled trial”

Supplementary Table 2. Ocular surface assessment parameters and methods.[1,2]

Assessment Parameter	Method (s)	Short-term assessment	Long-term assessment
Symptoms tests	• National Eye Institute Visual Function Questionnaire-25 (NEI-VFQ25)<br>• Ocular Surface Disease Index (OSDI)	✓	✓
Tear volume	• Schirmer's Test<br>• Phenol red thread test	✓	✓
Tear Film Stability	• Tear break-up time (TBUT):<br>• Fluorescein breakup time (FBUT)<br>• Non-invasive breakup time (NIBUT)	✓	✓
Tear osmolarity	• TearLab, I-PEN, LacriPen	✓	✓
Ocular Surface Irritation	• Fluorescein clearance test (FCT)	✓	
Corneal Epithelial Damage	• Ocular staining score with fuorescein	✓	✓
Conjunctival Hyperemia	• Ocular staining score with Lissamine green	✓	
Inflammation	• Tear biomarkers	✓	✓

Chronic Ocular Surface Inflammation	• Impression cytology (MC, IMC, IMB)<br>• Evaluation of conjunctival biomarkers (PCR, FC)		✓
Epithelial Barrier Dysfunction	• Impression cytology (MC, IMC, IMB)		✓
Ocular surface damage	• In-vivo confocal microscopy (IVCM)		✓
Calcium deposits	• Slit lamp examination<br>• Optical coherence tomography (OCT) scanning		✓
Ocular surface sensitivity	• Cochet-Bonnet Corneal Esthesiometer		✓

[1] Wolffsohn JS, Arita R, Chalmers R, Djalilian A, Dogru M, Dumbleton K, Gupta PK, Karpecki P, Lazreg S, Pult H, Sullivan BD, Tomlinson A, Tong L, Villani E, Yoon KC, Jones L, Craig JP. TFOS DEWS II Diagnostic Methodology report. Ocul Surf. 2017 Jul;15(3):539-574

[2] Willcox MDP, Argüeso P, Georgiev GA, Holopainen JM, Laurie GW, Millar TJ, Papas EB, Rolland JP, Schmidt TA, Stahl U, Suarez T, Subbaraman LN, Uçakhan OÖ, Jones L. TFOS DEWS II Tear Film Report. Ocul Surf. 2017 Jul;15(3):366-403.

Abbreviations: FC, flow cytometry; IMB, immunoblotting; IMC, immunocytochemistry; MC, microscopy; PCR, polymerase chain reaction
